# Endocrine modulation of cortical and retinal baseline perfusion across the menstrual cycle

**DOI:** 10.1177/0271678X261421106

**Published:** 2026-02-16

**Authors:** Melissa E Wright, Ian D Driver, Cassandra Crofts, Saajan Davies, Hannah Chandler, Michael Germuska, Ylenia Giarratano, Darwon Rashid, Miguel O Bernabeu, Louise Terry, Jessica J Steventon, Kevin Murphy

**Affiliations:** 1 School of Physics and Astronomy, Cardiff University Brain Research Imaging Centre (CUBRIC), Cardiff University, Cardiff, UK; 2 Department of Radiology, University of California Davis Medical Center, Sacramento, CA, USA; 3 Usher Institute, Centre for Medical Informatics, The University of Edinburgh, Edinburgh, UK; 4 School of Optometry and Vision Sciences, Cardiff University, Cardiff, UK; 5 School of Medicine, Cardiff University Brain Research Imaging Centre (CUBRIC), Cardiff University, Cardiff, UK

**Keywords:** Cerebrovasculature, neuroimaging, OCT, oestrogen, progesterone

## Abstract

The ovarian hormones, oestrogen and progesterone, have vaso- and neuroprotective effects, potentially due to cerebrovascular interactions. This study investigates their neuroendocrine influence on cerebral and retinal baseline vascular metrics across a healthy menstrual cycle. Twenty-six menstruating females completed imaging sessions and assessment of circulating hormone levels during their early follicular, late follicular and mid-luteal phase. Perfusion, arterial arrival time (AAT), global oxygen extraction fraction (OEF), cerebrovascular metabolic rate of oxygen (CMRO_2_), carotid artery radius and carotid pulsatility index (PI) were measured using 3 T MRI. Retinal vessel density and blood flow resistance were assessed with optical coherence tomography angiography (OCT-A). Assessed with linear models, increased oestradiol was related to increased global perfusion (χ^2^ (1) = 91.623; *p* = 1.049 × 10^−21^) and decreased AAT (χ^2^ (1) = 106.950; *p* = 4.575 × 10^−25^). An independent progesterone increase was also associated with increased global perfusion (χ^2^ (1) = 19.512; *p* = 9.998 × 10^−6^) and decreased AAT (χ^2^ (1) = 40.062; *p* = 2.46 × 10^−10^). A relationship was also found between increased oestradiol and decreased retinal blood flow resistance (χ^2^ (1) = 5.28; *p* = 0.0215), primarily driven by centrally localised vessels. This study finds that circulating oestrogen increases blood flow in the eye and brain, while progesterone is associated with brain perfusion increases alone. Both are associated with decreased cortical blood arrival speed. These effects suggest a potential pathway for neuroprotective mechanisms.

## Introduction

Ovarian hormones, such as oestrogen and progesterone, are considered to have vaso- and neuro-protective effects. A longer reproductive lifespan, and therefore a greater lifetime exposure to oestrogen, is associated with lower cardiovascular disease and dementia risk,^1–4^ greater cortical volume^5,6^ and greater later-life cognitive performance.^7^ Events associated with a decline in oestrogen levels (e.g. menopause) demonstrate an increased risk of vascular pathology, dementia and glaucoma.^8–13^ Progesterone is also shown to be protective against progression in a range of animal disease models (e.g. dementia, multiple sclerosis; for a review, see Bassani et al.^14^). A retrospective study of a UK national database found that irregular menstrual cycles (variation >20 days, as defined by clinical records) show an association with increased incidence of cardio- and cerebrovascular disease during a 26-year follow up.^15^ Considering the vasodilatory influence of oestrogen reported in animal models,^16,17^ endocrine modulation of the cerebrovascular system may contribute to this protective effect, especially as oestrogen receptors are found throughout the brain and retinal layers.^18–22^ Furthermore, modulation of the vascular system is observed following introduction of external hormone manipulation (e.g. hormonal contraceptives^23^ or gender-affirming hormone replacement therapy^24,25^). However, the influence of endocrine states on the human cerebrovascular system remains poorly understood.

The menstrual cycle provides an ideal model to investigate the influence of endogenous ovarian hormones on the cerebrovasculature. Across a menstrual cycle, levels of oestrogen and progesterone demonstrate relatively predictable fluctuations that can be utilised to investigate how acute high and low hormone states impact cerebrovasculature. Menstrual hormones have been found to modulate cortical volume (particularly medial temporal structures),^26–31^ estimates of brain age,^32^ neurotransmitter levels^33–35^ and resting/task-related cortical function.^36–41^ These cortical changes may also contribute to menstrual symptoms.^42–44^ To place these results in context and to understand the interactions between ovarian hormones and the neural system, it is important to also fully profile cerebrovascular physiology across this time period.

Early research using transcranial Doppler ultrasound suggests that blood flow metrics differ in the luteal phase, in which both oestrogen and progesterone are elevated.^45^ Otomo et al.^46^ investigated differences in quantitative perfusion using pseudo-continuous arterial spin labelling (pCASL) MRI methods and a sample of eight menstruating female participants. They reported an increase in frontal pole perfusion in the follicular phase compared to the luteal phase; however, without serum hormone measurements or more detailed information about test days, it is difficult to make conclusions about the specific neuroendocrine mechanisms. Cote et al.^47^ also investigated the endocrine modulation of perfusion across a menstrual cycle and found oestrogen and progesterone demonstrated opposing and regionally distinct influences. These studies begin to illustrate the impact of menstrual-related hormones on brain vasculature.

The retina represents a more easily imaged section of the central nervous system than the brain, and structural changes have been identified as independent predictors for a range of systemic diseases.^48^ Optical coherence tomography angiography (OCT-A), which allows for the visualisation of the vessels that permeate and nourish the retina, is highly complementary to vascular MRI. OCT-A has been utilised to demonstrate a decrease in vessel density in the ovulatory phase of the menstrual cycle, compared to the follicular and luteal phases.^49^ However, circulating hormone metrics were not collected to allow for mechanistic inference. Recent OCT-A analysis frameworks extract additional informative vascular metrics, including blood flow resistance, to enable a more comprehensive characterisation of retinal vessel properties.^50,51^ Overall, the literature illustrates short-term, menstrual-related changes on the cerebrovascular system, but this has yet to be investigated with a detailed cerebrovascular battery across brain and eye.

The current study thoroughly catalogues how a range of baseline cerebral and retinal vascular metrics vary with menstrual-related endocrine fluctuations in oestradiol and progesterone. Oestradiol is the most potent form of oestrogen and the form most commonly found in reproductive age women, showing regular fluctuations across a menstrual cycle.^52^ Such a thorough investigation is vital to gain a full picture of the influence of hormones on vascular health, place previous results in better context and highlight possible mechanisms behind menstrual-related symptoms, such as migraine.

Participants were tested at three time points; the early follicular phase (EFP; where oestradiol and progesterone are at ‘baseline’), the late follicular phase (LFP; where progesterone should remain low but oestrogen peaks) and the mid-luteal phase (MLP; where both hormones are expected to be elevated). As an exploratory aim, we investigated how vascular metrics across brain and eye relate to each other and how this coupling differs across high and low endocrine states.

This study therefore has the following aims:

1. Determine the contribution of circulating oestradiol and/or progesterone to variance in brain and retinal baseline vascular physiology measured across a healthy menstrual cycle.
2. Determine if the relationships between the outcome vascular metrics measures differ between high and low endocrine conditions.

## Methods

Participants were tested three times across a menstrual cycle, according to the self-reported onset day of menses. The three testing periods were the early follicular phase (EFP; scanned within days 1–4 of a cycle), the late follicular phase (LFP; 10–12 days) and the mid-luteal phase (MLP; 20–22 days). These days were defined as days from the onset of the last menstrual period, based on self-report. Participants fasted for a minimum of 4 h before testing and all sessions took place at a similar time of day. Participants were also asked to abstain from caffeine, alcohol and strenuous physical activity 12 h before testing sessions, to avoid any influence these may have on haemodynamics.^53^ Each testing session comprised of blood sampling, an MRI scan and an ocular imaging session.

A summary of outcome variables is presented in Table 1.

**Table 1.** All vascular outcome variables.

Outcome variable	Methodology	Region of interest
Perfusion	MRI – MPLD–pCASL	Desikan–Killiany Atlas (41 regions/hemisphere)
AAT	MRI – MPLD–pCASL	Desikan–Killiany Atlas (41 regions/hemisphere)
OEF	MRI – TRUST	Global (via sagittal sinus)
Global CMRO_2_	MRI – TRUST and MPLD–pCASL	Global (via sagittal sinus)
Carotid radius	MRI – TOF	Carotid arteries (left and right)
PI	MRI – DIMAC	Carotid arteries (left and right)
Retinal vessel density	OCT-A	Central fovea, parafovea (left and right eyes)
Retinal blood flow resistance	OCT-A	Central fovea, superior, temporal, nasal, inferior (left and right eyes)

AAT: arterial arrival time; CMRO_2_: cerebral metabolic rate of oxygen; MRI: magnetic resonance imaging; MPDL–pCASL: multi post labelling delay pseudocontinuous arterial spin labelling; TRUST: T_2_-relaxation-under-spin-tagging; DEXI–pCASL: dual-excitation pseudocontinuous arterial spin labelling; TOF: time of flight; DIMAC: dynamic inflow magnitude contrast; OCT-A: optical coherence tomography angiography; OEF: oxygen extraction fraction; PI: pulsatility index.

### Participants

Twenty-six menstruating participants (age mean (SD) = 22.98 (3.58) years) were recruited. An initial screening session determined eligibility and sought written informed consent. Eligible participants had a regular (i.e. self-reported cycle length between 27 and 31 days for at least the last three cycles) menstrual cycle. Participants were excluded if they had any commonly accepted contraindications to MRI scanning, a clinically significant condition (such as diabetes or a neurological, cardiovascular, psychiatric, cerebrovascular or respiratory condition), were pregnant or had been in the last 6 weeks, took hormonal contraceptives presently or in the last 6 months, were post-menopausal, recent history (last 2 years) of self-reported menstrual irregularities (e.g. variable cycle length or frequency), menstrual cycle-related disorder (e.g. polycystic ovary syndrome (PCOS), dysmenorrhea), demonstrated an intraocular pressure of >21 mmHg (assessed before ocular imaging via an iCare IC100 Tonometer, Mainline Instruments LTD., UK), or had an ocular condition that may impact measurements (e.g. glaucoma or macular disease). Additionally, participants were required to complete a COVID-19 screening questionnaire before each session. Testing sessions were rearranged if participants reported any COVID-related symptoms, and participants were excluded if they were clinically vulnerable or extremely vulnerable to COVID-19. Participants received financial compensation for completed testing sessions. Ethical approval was given by the Cardiff University School of Medicine Research Ethics Committee and was in accordance with the Declaration of Helsinki.

Due to factors such as participant drop out and equipment availability, not all participants contributed to every timepoint and methodology. A breakdown of sample sizes is given in Table 2. All sizes meet our calculated sample size threshold for 90% power (*n* = 14 required based on effect size dz = 0.98 in ΔMCAv (middle cerebral artery velocity)^54^).

**Table 2.** Breakdown of participant sample sizes contributing to each timepoint and methodology.

Sample	Total	EFP	LFP	MLP
Overall	26	22	22	23
MRI	21	17	17	18
OCT-A	25	20	19	20

EFP: early follicular phase; LFP: late follicular phase; MLP: mid-luteal phase; MRI: magnetic resonance imaging; OCT-A: optical coherence tomography angiography.

OCT-A scans were taken in both left and right eyes in each participant.

### Hormone sampling

To assess circulating hormone levels, 5 ml of blood was sampled via venepuncture from the hand/arm. The anonymised blood sample was sent to the Medical Biochemistry Laboratories at the University Hospital of Wales for measurement of oestradiol and progesterone serum concentration as NHS standard. Oestradiol levels were measured with an Abbott Architect analyser, while progesterone levels were measured with an Abbott Alinity analyser.

Intercorrelation between these hormones reduces the power of the linear models used; therefore, progesterone values were transformed. Oestradiol levels were regressed from progesterone levels and the model residuals were taken as the variance of progesterone, that is, independent from oestradiol, referred to as ‘resProgesterone’.

### MRI session – Acquisition

Data were acquired at Cardiff University Brain Research Imaging Centre (CUBRIC) on a Siemens MAGNETOM Prisma 3T scanner with a 32-channel head coil. A high-resolution structural T1 image was collected via an MPRAGE scan (1 mm^3^; repetition time (TR) = 2.1 s; echo time (TE) = 3.24 ms; inversion time (TI) = 850 ms). The structure of cerebral arteries was imaged using a time-of-flight (TOF) scan (TR = 21 ms; TE = 3.34 ms; FA = 18; GRAPPA = 3; eight slabs; voxel size = 0.6 mm^3^).

Global oxygen extraction fraction (OEF) was assessed using a T_2_-relaxation-under-spin-tagging (TRUST)^55,56^ sequence (TR = 3 s; TE = 3.9 ms, effective TEs = 0, 40, 80, 160 ms). This was manually positioned over the sagittal sinus, using the AC–PC and occipital notch as navigational anatomical landmarks. A T1 inversion recovery sequence (ΔTR/TE = 150/22 ms, flip angle = 90° and GRAPPA acceleration factor = 2; with 960 acquisitions >16 repeats of 60 measurements) was also collected to quantify the T1 of venous blood. This was used to calculate haemoglobin (Hb),^57^ necessary for estimating OEF and then CMRO_2_ (cerebral metabolic rate of oxygen). An in-house multi post-labelling delay pseudocontinuous arterial spin labelling (MPLD–pCASL) perfusion scan was completed to assess perfusion and arterial arrival time (AAT), with the tagging plane positioned perpendicular to the internal carotid arteries using the TOF scan as reference (maximum TR = 5.6 s; TE = 11 s; voxel resolution = 3.4 × 3.4 × 6.0 mm; tag duration = 1500; post-labelling delays (PLDs) = 250–3000 ms in steps of 250 ms; GRAPPA = 2). Siemens in-built prescan normalise correction was applied to remove spatial signal inhomogeneity due to receive array coil sensitivity. To allow quantification, two separate equilibrium magnetisation maps (M0 scan; phase encoding direction PA and AP) were also obtained, with TR = 6000 ms and TE = 11 ms.

Pulsatility index (PI) in the internal carotid arteries was measured using dynamic inflow magnitude contrast (DIMAC) imaging.^58^ A fast single-slice echo planar imaging (EPI) image was acquired, which suppresses static signal leaving the inflowing blood signal in the flow-velocity regime.^58,59^ This provided flow-velocity-weighted images with a 15 ms temporal resolution, allowing the pulse waveform to be resolved for individual heartbeats. The single EPI slice was acquired perpendicular to the internal carotid arteries (TR = 15 ms; TE = 6.8 ms; flip angle = 90°; 2 × 2 × 10 mm; 200 × 200 mm field of view; GRAPPA 5; phase partial Fourier 0.75; 4096 repetitions in 65 s; slice thickness = 10 mm).

To monitor and measure physiological data during the scan, a pulse oximeter (Biopac Systems, Inc., CA, USA), nasal cannula (AD Instruments gas analyser) and custom-built respiratory belt were secured.

### MRI pre-processing

#### Cerebral blood flow and arterial arrival time

Pre-processing of the pCASL sequence was completed using the Analysis of Functional NeuroImages (AFNI) software package^60^ (provided in the public domain by the National Institutes of Health, Bethesda, MA, USA; http://afni.nimh.nih.gov/afni). Following motion correction, the scan was split into separate PLDs (five pairs of each) and the tag minus control signal change in magnetisation was calculated for each. For quantification, the *M*_0_ of blood was calculated as:

$$M_{0}blood=\frac{M_{0}csf*\exp(TE*\left(\left(\frac{1}{T_{2}csf}\right)-\left(\frac{1}{T_{2}blood}\right)\right))}{\lambda csf}$$

λcsf = blood–CSF partition coefficient (1.15)^61,62^

$M_{0}csf$ = the *M*_0_ of the cerebral spinal fluid (CSF) was taken from a CSF mask generated around a manually positioned central point in the lateral ventricles.

TE = echo time (0.019 s)

$T_{2}csf$ = T_2_* of CSF (0.4)^61^

$T_{2}blood$ = T_2_* of blood (0.06s)^63^

Using a non-linear fitting algorithm (AFNI’s 3dNLfim function), the general kinetic perfusion model provided final quantification.^64^ Final perfusion and AAT maps were thresholded by a goodness-of-fit measure (*R*^2^ > 0.6) to remove spurious model fits.

FLIRT^65^ was used to generate a transformation matrix to register the Desikan-Killiany Atlas to each participants’ native space. The cortical regions were further restricted to the individual’s grey matter voxels (for more information, see Supplemental Material). The median perfusion and AAT were extracted for each region from voxels that passed threshold.^66^ The average perfusion and AAT across all surviving voxels were taken as global measures for comparison.

#### Global oxygen extraction fraction and cerebral metabolic rate of oxygen

Global OEF and CMRO_2_ metrics were estimated using TRUST scan data and global grey matter perfusion values estimated from the PCASL data and a grey matter mask. The T_2_ of blood was calculated by non-linear least squared fitting of a mono-exponential equation to the TRUST difference data, as a function of the effective echo times and T_2_. The average of the two most intense voxels from a sagittal sinus regions-of-interest (ROI) was used and venous oxygenation (Yv) was estimated by inverting the relationship between Yv, Hb and the T_2_.^55^ OEF was calculated as:

$$OEF=\frac{\left(SaO_{2}-SvO_{2}\right)}{SaO_{2}}$$

Global CMRO_2_ was calculated as:

$$CMRO_{2}=gmCBF*CaO_{2}$$

where CaO_2_ is defined as:

$$CaO_{2}=\left(\left[Hb\right]*1.34*\;\;SaO_{2}\right)*OEF*39.34$$

SaO_2_ was assumed to be 98% as this was a healthy young cohort.

#### Time-of-flight analysis

TOF scans were analysed to obtain the average radius of the carotid arteries. Using the Vascular Modelling Toolkit (VMTK),^67^ the ROI, defined as the length of artery between the carotid scan slice position and the first branch of the middle cerebral artery, was extracted and converted into a 3D surface projection. Centrelines were plotted and the average vessel radius was calculated by plotting a Voronoi diagram of the closest points on the surface projection to each point on the centreline. The carotid radius was extracted for the left and right side separately. The surface model was separated into two halves and the carotid arteries in each surface were manually separated from the halves of the Circle of Willis. Non-carotid vessels or vessel fragments were then removed using VMTK’s surface connectivity function, which removes all structures except the largest in the model. When either carotid could not be isolated due to noise, only one artery was included in the analysis (EFP = 2, LFP = 1, MLP = 2). When data for both carotid arteries were not sufficiently high quality to extract the carotid surface, that participant was excluded (EFP = 3, LFP = 5, MLP = 4).

#### Carotid pulsatility index

Left and right internal carotid artery DIMAC timeseries were formed by averaging over a 4 × 4 voxel square region, centred on each artery. A 3 s cut-off high-pass filter and fifth-order (21 timepoint frame length) Savitzky–Golay low-pass filter was applied to each timeseries, then each pulse period was modelled by a Fourier series basis set, with five sine/cosine pairs with periods ranging from one to one fifth of the pulse period. PI was calculated as the range of the modelled timeseries divided by the mean for each pulse period, then averaged across all pulse periods in the scan.

### OCT session acquisition

OCT-A images were acquired on a Triton Swept-Source OCT device (TOPCON healthcare (Great Britain) Medical Limited, Newbury, UK). A fovea-centred OCT-A scan was acquired in both eyes (scan area = 3 × 3 mm).

Participants were instructed to fixate on a central cross and remain as still as possible. At least two images were taken in each eye, with at least a 60/100 quality rating (calculated by the instrument). These two images underwent manual subjective review by author MEW and the best quality scan, as defined by a lack of artefacts such as motion or blurring, was taken forward for further analysis. For one participant (all three phases), no OCT-A scans of sufficient quality were available, so they were excluded from the OCT-A analysis. For one timepoint in one participant, only the left eye provided sufficient image quality (one MLP).

Eye-tracking was enabled to minimise the contribution of eye movements. All images were obtained with minimal room lighting to ensure maximum natural pupil size.

### OCT-A pre-processing

**Figure 1.**
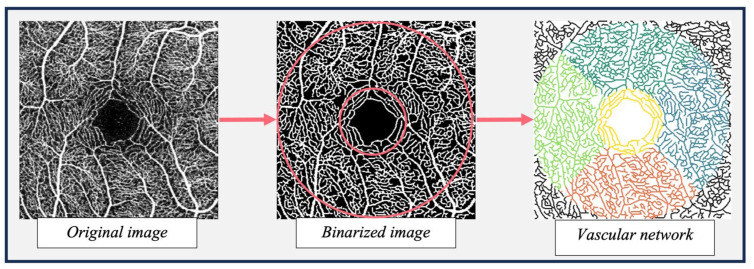
Illustration of the OCT-A analysis pipeline. Red rings = fovea (1 mm diameter) and parafovea (3 mm diameter) areas used to extract vessel density metrics. Coloured area = fovea, temporal, superior, nasal and inferior regions used for extracting retinal blood flow resistance metrics.

Vessel density and retinal blood flow resistance were estimated. Initially, a 2D image of the superficial retinal layer (from the internal limiting membrane layer to the inner plexiform layer, as defined by the instrument) was extracted and binarised to produce an image comprised of vessel (white) and non-vessel (black) pixels (see Figure 1).

This binarised image was used as the input to a bespoke analysis software. A detailed explanation of the software has been provided previously.^50,51^ In brief, the scans undergo automated segmentation using the optimally oriented flux vessel enhancement filter, followed by thresholding.^68^ Post-processing cleaning was performed to remove segmentation artefacts (i.e. small, isolated clusters of white pixels <30 pixels). The vasculature captured in the binarised image was then skeletonised and modelled as a network with nodes representing white pixels along the vessel centreline and edges connecting neighbouring pixels. The network was embedded into a Euclidean space and each node associated with the corresponding coordinates and vessel radius – distance between pixel in the centreline and the closest non-vessel pixel. Features were then extracted from the binary mask and the vascular network. The ratio of vessel pixels to overall number of pixels in the region of interest was extracted as the vessel density in the central fovea and parafovea (see Figure 1), whereas retinal blood flow resistance was calculated using Poiseuille’s law at each vessel segment as follows:

$$R\left(l,r\right)=\frac{\Delta P}{Q}=\frac{8\eta l}{\pi r^{4}}$$

∆*P* = pressure difference between two ends of a vascular segment,

*Q* = flow rate

*l* = length

*r* = radius

The constant viscosity of blood is assumed to be:

$$\eta =2.084\;\;x\;10^{-3}Pa\;\cdot s$$

The mean blood flow resistance was extracted for each ROI (the central fovea and superior, temporal, inferior and nasal regions; see Figure 1) and taken forward for further analysis.

### Statistical analysis

The contribution of circulating hormone levels to the variance in the outcome variables was calculated using linear mixed models. Statistical analysis was completed in R software (v.4.2.2)^69^ and linear models were constructed using the ‘lmerTest’ analysis package.^70^ Statistical significance was set at the *p* < 0.05 level. With all models, residual plots were visually inspected and showed no notable deviations from homoscedasticity or normality. Oestradiol, progesterone and ROI (if applicable) were used as fixed effects, with participant as a random effect. If a statistically significant main effect of hormone was found, an interaction term between ROI and hormone levels was added to investigate if the effect is driven by a particular ROI (if applicable). In all cases where there are more than two ROIs, the average value over all ROIs was included as a global comparison. For OCT-A and carotid metrics, both eyes/vessels were included in the analysis. The laterality (left or right) was inputted as a fixed effect in order to account for similarity between eyes/vessels.

The goal of the second aim was to investigate the relationships between vascular outcomes (detailed above; see Table 1) and investigate how these relationships differ by low or high hormonal state. To address this exploratory aim, the highest and lowest hormone phase for each participant was identified (if two phases had the same level, the earlier phase (i.e. EFP > LFP > MLP) was used for the low hormone condition and the later phase (i.e. MLP > LFP > EFP) was used for the high hormone condition). High and low groups for oestradiol and progesterone were grouped separately, leading to four conditions. The data were standardised and a cross-correlation matrix generated (Pearson’s rho). A principal components analysis (PCA) was used on each correlation matrix to reduce the dimensionality of the data and investigate shared variance across the variables.

## Results

### Hormones

**Figure 2.**
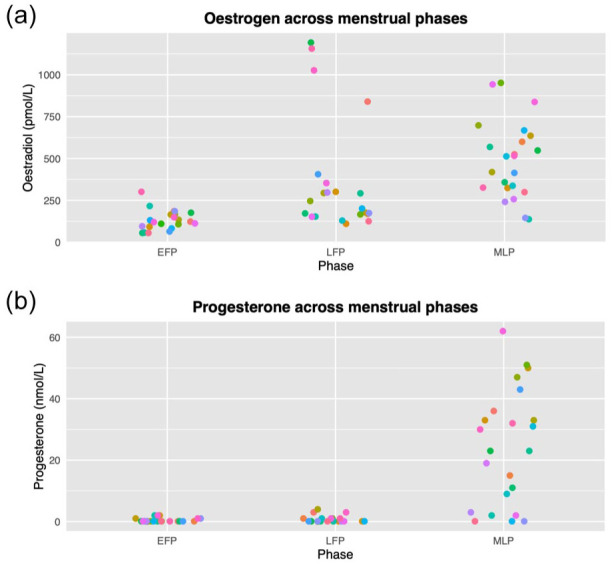
Levels of circulating hormones across the three tested menstrual cycle phases: (a) oestrogen (in the form of oestradiol (pmol/L)) and (b) progesterone (nmol/L). Coloured by individual participant. EFP: early follicular phase; LFP: late follicular phase; MLP: mid-luteal phase.

The levels of circulating oestradiol and progesterone for all participants are presented in Figure 2. The expected LFP oestrogen peak was not captured in all participants and there was a statistically significant correlation between levels of circulating oestradiol and progesterone (all participants and timepoints included; *r* (65) = 0.463; *p* = 7.95 × 10^−5^). Progesterone values were therefore transformed to avoid issues of intercorrelation. This value is referred to as ‘resProgesterone’.

### Cerebral blood flow

Oestradiol was found to explain a significant amount of global perfusion variance (χ^2^ (1) = 91.623; *p* = 1.049 × 10^−21^), with global perfusion increasing by 1.95 × 10^−2^ ml/100 g/min ± 2.02 × 10^−3^ (standard error (SE)) with each unit increase of oestradiol. This represents an average variation of 9.90 ml/100 g/min across a menstrual cycle (based on the average max-min of hormone level in participants with more than one timepoint (507 pmol/L), though previous literature suggests the fluctuation in oestradiol across a cycle can be up to 2× larger).^29,71,72^ Levels of resProgesterone also explained a significant amount of perfusion variance (χ^2^ (1) = 19.512; *p* = 9.998 × 10^−6^), with perfusion increasing by 0.17 ml/100 g/min ± 0.04 (SE) with each unit increase of resProgesterone, representing a change of 3.47 ml/100 g/min across a menstrual cycle (assuming a difference of 20.433 nmol/L resProgesterone).

Interaction terms between ROI and each set of hormone levels did not explain a significant amount of variance (i.e. the relationship between the hormone and perfusion in each ROI didn’t significantly differ from that globally; oestradiol: χ^2^ (83) = 91.123; *p* = 0.254; resProgesterone: χ^2^ (83) = 64.978; *p* = 0.928).

### Arterial arrival time

Oestradiol was found to explain a significant amount of global AAT variance (χ^2^ (1) = 106.950; *p* = 4.575 × 10^−25^), with global AAT decreasing by 2.44 × 10^−4^ s ± 2.34 × 10^−5^ (SE) with each unit increase of oestradiol. This represents an average variation of 0.12 s across a menstrual cycle. Levels of resProgesterone also explained a significant amount of variance (χ^2^ (1) = 40.062; *p* = 2.46 × 10^−10^), with AAT decreasing by 2.87 × 10^−3^ s ± 4.52 × 10^−4^ (SE) with each unit increase of resProgesterone, representing a change of 0.06 s across a menstrual cycle.

Interaction terms between ROI and each set of hormone levels did not explain a significant amount of variance (i.e. the relationship between the hormone and AAT in each ROI didn’t significantly differ from that globally; oestradiol: χ^2^ (83) = 77.338; *p* = 0.655; resProgesterone: χ^2^ (83) = 73.624; *p* = 0.760).

### Global oxygen extraction fraction

Neither oestradiol (χ^2^ (1) = 0.451; *p* = 0.502) nor resProgesterone (χ^2^ (1) = 0.381; *p* = 0.537) explained a significant amount of global OEF variance.

### Global cerebral metabolic rate of oxygen

As above, neither oestradiol (χ^2^ (1) = 2.629; *p* = 0.105) nor resProgesterone (χ^2^ (1) = 0.222; *p* = 0.637) explained a significant amount of global CMRO_2_ variance.

### Carotid radius

In the carotid radius model, one datapoint with an outlier model residual value (defined as 2.5 × SD away from the mean) was removed due violation of the model assumptions (linearity and heteroscedascity; further investigation found this datapoint also had an outlier radius value). Neither oestradiol (χ^2^ (1) = 0.017; *p* = 0.895) nor resProgesterone (χ^2^ (1) = 0.849; *p* = 0.357) explained a significant amount of carotid radius variance.

### Carotid pulsatility index (PI)

Residual plots of the carotid PI model were examined, and two datapoints excluded for outlier residual values that caused assumption violations. After examination of the hormone main effects, neither oestradiol (χ^2^ (1) = 0.758; *p* = 0.860) nor resProgesterone (χ^2^ (1) = 1.029; *p* = 0.794) explained a significant amount of carotid PI variance.

### Retinal vessel density

Neither oestradiol (χ^2^ (1) = 0.002; *p* = 0.964) nor resProgesterone (χ^2^ (1) = 0.216; *p* = 0.642) explained a significant amount of vessel density variance.

### Retinal blood flow resistance

Oestradiol was found to explain a significant amount of resistance variance (χ^2^ (1) = 5.283; *p* = 0.0215), decreasing resistance by −1.89 × 10^−8^ Pa · s/μm^3^ ± 8.11 × 10^−9^ (SE) with each unit increase of oestradiol. This translates to a change of blood flow resistance across the menstrual cycle of −1.2 × 10^−4^ Pa · s/μm^3^. The resProgesterone factor, however, did not significantly contribute to the model (χ^2^ (1) = 0.395; *p* = 0.530).

This interaction between oestradiol and ROI was significant (χ^2^ (5) = 11.603; *p* = 0.0407). By inspecting the individual fixed effects, the oestradiol-resistance relationship was found to be significantly greater in the central (foveal) region compared to the global average (*t* (6.765 × 10^2^) = −2.136; *p* = 0.033; estimate = −5.17 × 10^−8^; SE = 2.42 × 10^−8^), indicating that this region may be driving the result. The other ROIs did not significantly differ from the global average (nasal: *t* (6.765 × 10^2^) = 0.568, *p* = 0.571; inferior: *t* (6.765 × 10^2^) = 0.335, *p* = 0.738; temporal = *t* (6.765 × 10^2^) = 0.832, *p* = 0.406; superior: *t* (6.765 × 10^2^) = 0.402, *p* = 0.688).

A summary of results is provided in Table 3. Endocrine influences on haemoglobin levels and end-tidal CO_2_ were also examined (see Supplemental Material) but no statistically significant effects were found, suggesting this did not influence these results.

**Table 3.** Summary of results from all vascular outcome variables.

Outcome	Hormone	χ^2^ statistic	*p* value	Regional effect	Direction
Perfusion	Oestradiol	91.623	<0.001	n.s.	Increase
	Progesterone	19.512	<0.001	n.s.	Increase
AAT	Oestradiol	106.950	<0.001	n.s.	Decrease
	Progesterone	40.062	<0.001	n.s.	Decrease
OEF	Oestradiol	0.451	n.s.	Global	=
	Progesterone	0.381	n.s.	Global	=
CMRO^2^	Oestradiol	2.629	n.s.	Global	=
	Progesterone	0.222	n.s.	Global	=
Carotid radius	Oestradiol	0.017	n.s.	–	=
	Progesterone	0.849	n.s.	–	=
PI	Oestradiol	0.758	n.s.	–	=
	Progesterone	1.029	n.s.	–	=
Retinal vessel density	Oestradiol	0.002	n.s.	–	=
	Progesterone	0.216	n.s.	–	=
Retinal blood flow resistance	Oestradiol	5.283	<0.05	Yes	Decrease
	Progesterone	0.395	n.s.	–	=

n.s.: not significant.

### Exploratory relationships analysis

In an exploratory analysis, we investigated how the relationships between vascular metrics differ in high and low endocrine state. While this study was not designed to test this research question, this data can give an idea of whether there is a pattern in how low/high hormone states impact vascular relationships, which can then be specifically investigated in the future.

**Figure 3.**
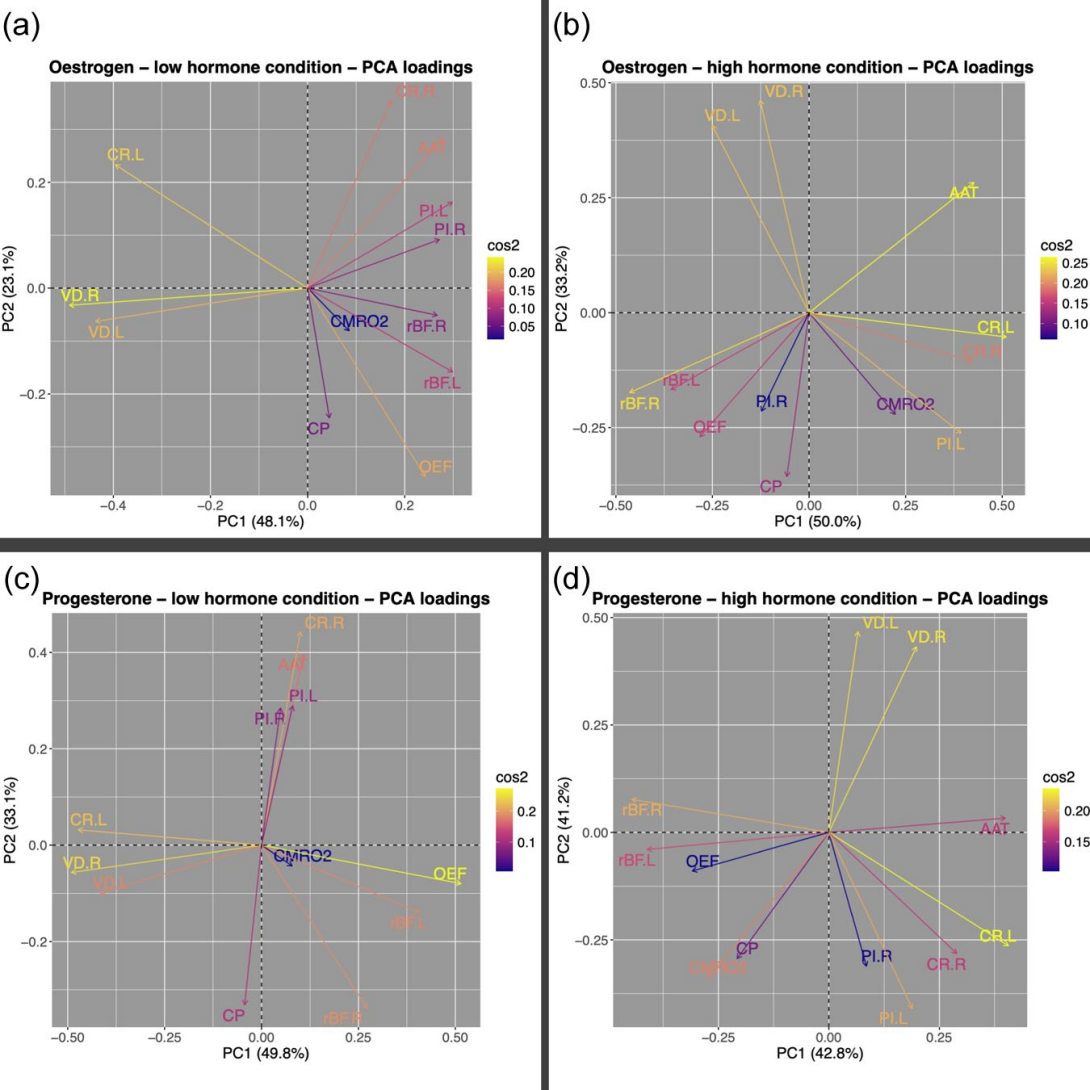
Loadings of all variables on the two extracted principal components (PC1 and PC2) following PCA of each condition: (a) low oestrogen, (b) high oestrogen, (c) low progesterone and (d) high progesterone. Cos2 refers to the importance of that component/dimension to that particular variable. AAT: arterial arrival time; CP: cerebral perfusion; CR.L: carotid radius left; CR.R: carotid radius right; CMRO_2_: cerebral metabolic rate of oxygen; OEF: oxygen extraction fraction; PI.L: pulsatility index left; PI.R: pulsatility index right; rBF.L: retinal blood flow resistance left; rBF.R: retinal blood flow resistance right; VD.L: retinal vessel density left; VD.L: retinal vessel density left; PCA: principal component analysis.

**Figure 4.**
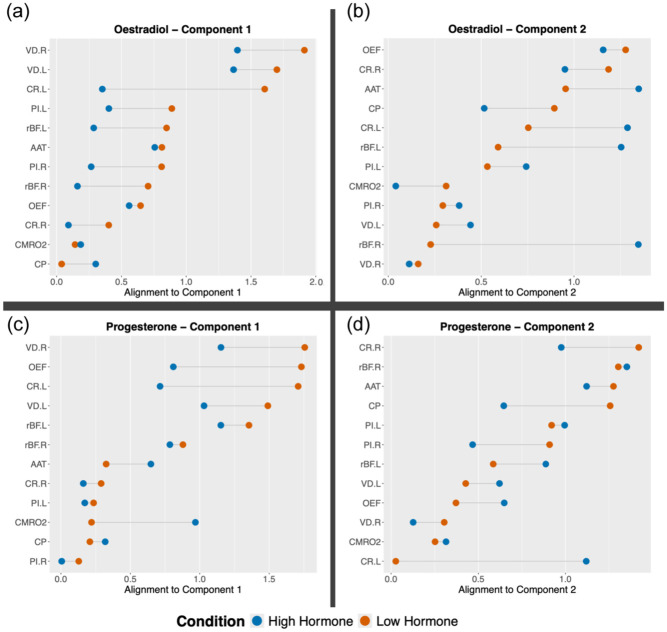
The amount to which each vascular outcome variable (from either the high or low hormone condition) is aligned with each principal component plotted for the oestradiol and progesterone conditions. Blue datapoints are from the high hormone condition (either oestradiol (a, b) or progesterone (c, d)), while orange datapoints are from the low hormone condition (either oestradiol (a, b) or progesterone (c, d)). AAT: arterial arrival time; CP: cerebral perfusion; CR.L: carotid radius left; CR.R: carotid radius right; CMRO_2_: cerebral metabolic rate of oxygen; OEF: oxygen extraction fraction; PI.L: pulsatility index left; PI.R: pulsatility index right; rBF.L: retinal blood flow resistance left; rBF.R: retinal blood flow resistance right; VD.L: retinal vessel density left; VD.L: retinal vessel density left.

PCA was completed on all conditions (low oestrogen, high oestrogen, low progesterone, high progesterone; cross-correlation tables and scree plots available in the Supplemental Material). In all conditions, the first two components explained at least 70% of the variance and the loadings of all variables onto these components were plotted. As can be seen from Figure 3, spreads were varied but the variables are slightly more clustered in the low hormone condition for oestrogen and progesterone than the high hormone condition, suggested that there is less shared variance in the high endocrine conditions. The data from the high hormone conditions were then projected to the principal component space generated by the corresponding low hormone condition. Again, the first two components were examined. The average amount to which each participant’s data aligned with each component was reduced in the high hormone condition by a small percentage in 3/4 components (Oestrogen component 1: +8.61%; oestradiol component 2: −11.84%; progesterone component 1: −3.08%; progesterone component 2: −9.07%; variable component alignment plotted in Figure 4). For the low oestrogen condition, there was roughly a greater contribution of most of the retinal metrics and PI in the first component, while the second component had greater loading from perfusion, carotid radius and metabolism (OEF, CMRO_2_). For the low progesterone components, roughly the retinal metrics and OEF showed greater contributions to the first component, while the others demonstrated greater loadings to the second.

## Discussion

This study investigated the neuroendocrine influence of menstrual hormones (i.e. oestrogen and progesterone) on a battery of baseline cerebrovascular and retinal vascular metrics. Animal research has suggested that oestrogen increases vasodilation and perfusion,^16,17^ maintains blood–brain barrier function,^73^ promotes angiogenesis^17^ and improves recovery processes after ischaemic injury,^74,75^ most of which are longer term processes. Progesterone has also been linked to improved functional outcomes (e.g. external rating of physical and cognitive functional status in humans or neurological behavioural score in rodent models) following brain injury,^76–78^ increased vascular reactivity^79,80^ and inflammation (though evidence for the direction of this effect differs).^81,82^ Early work has started to find variations in aspects of baseline vascular physiology sensitive to menstrual-related endocrine fluctuations,^45–47,49^ but this has yet to be investigated with a thorough resting cerebrovascular battery across both brain and eye. This is important to establish as changes in baseline cerebrovascular physiology will in turn influence the health of the surrounding neural tissue and may also interact with more dynamic vascular functions, such as vascular reactivity to a vasoactive substance (e.g. CO_2_) or neural activity, though this needs further research to establish.

Oestradiol and additional progesterone variance were modelled so that their individual contributions could be calculated. We found that the oestradiol regressor was significantly associated with increased perfusion, which is in line with previous animal and human research.^16,47^ Progesterone also significantly contributed to increased perfusion. The cumulative perfusion increase is ~12% across the menstrual cycle, which is notable when considering it occurs over the regular fluctuations of the menstrual cycle, which are relatively small hormonal changes compared to other key life changes, such as pregnancy or menopause. Contrary to previous data by Cote et al.,^47^ we found that these neuroendocrine effects occurred globally across the cortex. The exact mechanism underlying the observed increase in perfusion with hormone level requires further investigation. One possibility is that it may reflect changes in cortical vascular resistance, especially considering the association between oestradiol and reduced retinal blood flow resistance found in this sample.

The influence of hormones on baseline perfusion has a number of implications. Firstly, blood oxygen level dependant (BOLD) fMRI is a common technique for investigating neural activation. The BOLD signal is a complex phenomenon that reflects both neural activity and neurovascular coupling, with the final BOLD response reduced by global increases in baseline perfusion.^83,84^ Endocrine-dependant changes in perfusion may therefore lead to extra noise in studies with menstruating participants, especially if comparing groups or timepoints associated with different endocrine states (e.g. menstrual cycle phase, menopause). Measuring hormone levels would be an ideal way of accounting for and controlling for this factor, but controls could also be more easily implemented by asking the time since last period to account for menstrual phase or by testing during the EFP (when hormones are expected to be at ‘baseline’). Additionally, while this study investigated baseline resting cerebrovascular physiology, it is possible that hormone-related variations in baseline perfusion interact with other dynamic regulatory processes. Ovarian hormones have been associated with increased vasodilatory reactivity to hypercapnia and acetazolamide in both carotid and cerebral arteries,^85–87^ suggesting potential modulation of cerebrovascular responsiveness. Rapid fluctuations in blood pressure also influence moment-to-moment perfusion due to the latency in dynamic cerebral autoregulation. Although endocrine-related increases in baseline perfusion could theoretically shift the operating range of these regulatory mechanisms, acute changes in perfusion pressure would still be expected to produce measurable effects on perfusion. Existing studies indicate that autoregulatory efficiency^88,89^ and blood pressure^90^ are largely maintained across menstrual phases, implying that hormonal modulation of baseline perfusion may not substantially alter acute pressure-buffering mechanisms. Nevertheless, the interactions between baseline perfusion, vascular reactivity and autoregulatory function across the hormonal fluctuations of the menstrual cycle remain poorly understood and warrant targeted investigation. Finally, as perfusion changes can affect volumetric measurements, these results are also important to place menstrual-related investigations of cortical structure into better context. For example, as our observed neuroendocrine effects were global, it provides more confidence that regionally-specific menstrual changes to volume^26,28,30^ are driven by non-vascular factors.

Both ovarian hormones were also significantly associated with decreased global blood velocity (AAT), potentially due to an endocrine influence on vascular tone and increased vascular stiffness in cortical microvasculature. Previous literature on the endocrine relationship on systemic arterial stiffness (e.g. pulse wave velocity, augmentation index) has suggested a small decrease across the menstrual cycle^91^ and increase with exogeneous hormones (e.g. hormonal contraceptives),^92,93^ but the evidence is inconsistent.^93,94^ The context of endocrine state, vessel type and location are likely to be important factors in understanding this relationship.

For the other cortical measures in this study, we found no statistically significant effect of menstrual hormone fluctuations, suggesting that, across the menstrual cycle at least, these metrics are robust to hormone changes to the limit of our measurement sensitivity. For carotid radius, this replicates previous findings,^47^ but for OEF, CMRO_2_ and PI, this study represents the first demonstration of stability across the menstrual cycle, possibly reflecting that cerebral oxygen metabolism may be robust to acute fluctuations in menstrual hormones.

Regarding the retinal results, contrary to a previously found negative association,^49^ we found no significant interaction between menstrual hormones and vessel density across the retina. However, it should be noted that Guo et al.^49^ compared vessel density to menstrual phase rather than hormones, a slightly different comparison that inherently assumes hormone levels. It could be that these observed differences between phases were due to changing hormone levels, rather than the measured hormone level per se. For example, Guo et al. ensured that they tested during the ovulatory phase by monitoring luteinising hormone (LH) levels. The day following the peak was defined as the start of the ovulatory phase. However, the oestrogen peak has been observed to occur, most frequently, 2 days before ovulation.^95^ Our results together could therefore indicate that retinal vessel density is sensitive to fluctuating hormone levels (before regulatory mechanisms initiate) rather than the level of hormone itself at the time of testing. This hypothesis could be investigated by utilising high-density sampling across the menstrual cycle, which allows for hormonal changes to be better characterised and has recently been employed in neuroendocrine research.^96^ Alternatively, and given that OCT-A visualises vascular structure by detecting erythrocyte motion, it would be tempting to speculate that the changes in vessel density reported by Guo et al. are indeed indirect measurements of a potential change in retinal microvascular flow, in the absence of independent evidence of microvascular remodelling over the course of the menstrual cycle.

Finally, we found that oestrogen significantly decreased retinal blood flow resistance, which suggests that the effect on microvascular perfusion found in the cortex also extends to the retina. A regional analysis found that this result may be driven by the foveal vessels, potentially due to the smaller vessels present. If oestrogen has no impact on larger retinal vessels (or there is a more subtle relationship, as suggested with retinal vessel density), this may overshadow a small effect in the other ROIs. In addition, it is possible that arteries and veins behave differently and since we are not discriminating between them, their effect is balanced out in other ROIs.

As an exploratory aim, we examined whether relationships between the vascular metrics differed between low and high hormonal state. We investigated loadings onto PCA components and found a pattern of reduced commonality between the vascular metrics in the high endocrine condition compared to the low endocrine condition, possibly suggesting that the inter-function relationships were disrupted in high hormone levels. This may reflect the action of increased hormone levels on some metrics more than others, leading to a breakdown in their relationships. This change in relationship may also suggest a more subtle menstrual cycle-related effect, that is, not captured by the linear models that use hormone value alone; it may be in response to a change in endocrine state, or a different menstrual-related hormone. However, it is important to note that as an exploratory aim, the current study was not designed to be statistically powered for this type of research question and requires specific investigation in future work.

An overarching research interest is the potential protective effect of ovarian hormones for neurodegenerative conditions such as dementia and glaucoma.^1,3,9,10,12,13,97^ While the current study does not address this question directly, it does demonstrate an interaction between these hormones and the cerebrovascular system. Multiple studies provide strong evidence that vascular alterations across the eye and brain are associated with dementia or glaucoma risk/diagnosis.^98–106^ It may be argued that recurrent episodes of cerebral vasodilation and reduced vascular resistance induced by menstrual-related fluctuations in oestrogen and progesterone are associated with long-term benefits for the brain and cerebrovascular ageing. Indeed, previous work has reported lower cardiovascular and cerebrovascular risk with increased reproductive lifespan,^2,4^ presumably due to an increased number of menstrual cycles and associated fluctuations across the lifespan. This beneficial effect is also supported by lower heart rate variability being associated with increased white matter hyperintensities and cognitive impairment.^107–109^ This may therefore highlight that a regular endocrine influence on the cerebrovascular system may underlie a neuroprotective effect in later life. However, this is speculative and requires further investigation.

These results also have implications for other groups that show notable changes in endocrine state, including natural or surgical menopause, pregnancy, puberty, conditions with altered hormone levels (such as polycystic ovary syndrome) and hormone replacement therapy. Many of these states demonstrate greater changes in baseline hormone level than across a regular healthy menstrual cycle, so may also show greater changes in aspects of cerebrovascular physiology. The current study also raises questions about the impact of irregular menstruation, which can be associated with altered hormone levels. Indeed, many of these states and conditions are associated with changes in cardio- and cerebro-vascular disease risk.^110–114^

One of the limitations of the current study was that, although the phase days were chosen based on previous literature,^115^ the ovulatory oestrogen peak was missed in multiple participants (see Figure 2), which may have led to an underestimation of oestrogen’s influence. The variation in oestrogen menstrual pattern within and across people has been reported previously^95,116,117^ and is an important consideration when using methodologies such as MRI that may afford less scheduling flexibility. This highlights the importance of testing the actual circulating hormone level, rather than making assumptions based on menstrual phase, as this allows direct comparisons to the current hormone level. Additionally, by testing at least three times across a cycle, we were able to capture variation in both oestradiol and progesterone. In order to better investigate these changeable fluctuations in hormones, high-density sampling could be used, in which a smaller number of participants are densely sampled across the cycle to better characterise hormonal fluctuations. Alternatively, to better target phases of interest, ovulation tests could be employed. Finally, an important limitation is that blood pressure was not monitored. However, previous research suggests that the influence of menstrual cycle phase on haemodynamics is negligible, with no significant interaction with blood pressure.^90^ Additionally, the potential mediating influence of haemoglobin levels and end-tidal CO_2_ on endocrine-cerebrovascular relationships in this study was considered. When statistically examined, neither showed associations with hormone level (see Supplemental Material), suggesting they do not account for our results.

## Conclusion

In conclusion, we found that menstrual-related oestradiol and progesterone changes led to increased resting perfusion across brain and eye and decreased blood arrival time. This has important implications for menstrual-associated symptoms such as menstrual migraine, as well as cerebrovascular function in other endocrine states. By demonstrating that natural fluctuations in sex hormones interact with the cerebrovascular system, this also suggests that their neuro- and vaso-protective effect may be due to action on this system but requires further specific investigation.

## Supplemental Material

sj-docx-1-jcb-10.1177_0271678X261421106 – Supplemental material for Endocrine modulation of cortical and retinal baseline perfusion across the menstrual cycle Supplemental material, sj-docx-1-jcb-10.1177_0271678X261421106 for Endocrine modulation of cortical and retinal baseline perfusion across the menstrual cycle by Melissa E Wright, Ian D Driver, Cassandra Crofts, Saajan Davies, Hannah Chandler, Michael Germuska, Ylenia Giarratano, Darwon Rashid, Miguel O Bernabeu, Louise Terry, Jessica J Steventon and Kevin Murphy in Journal of Cerebral Blood Flow & Metabolism
