# Supplementary material for: Endocrine modulation of cortical and retinal baseline perfusion across the menstrual cycle
Source: J Cereb Blood Flow Metab. 2026 Feb 16:0271678X261421106. Online ahead of print. doi: 10.1177/0271678X261421106 (PMC12913051; doi:10.1177/0271678X261421106)
Supplement: sj-docx-1-jcb-10.1177_0271678X261421106 – Supplemental material for Endocrine modulation of cortical and retinal baseline perfusion across the menstrual cycle [file sj-docx-1-jcb-10.1177_0271678X261421106.docx]

**Supplemental material**

**Methods**

*Grey matter mask generation*

The T1 MPRAGE structural images were processed using the fsl_anat pipeline (1–3), which includes reorientating to standard orientation, bias-field correction, brain extraction, and tissue-type segmentation (FAST; 4). This generated a grey matter mask to be used to mask cortical atlas structures and grey matter perfusion.

**Results**

*Haemoglobin levels*

It was separately investigated whether the estimated Hb values used within the OEF and CMRO_2_ calculation were independently influenced by hormone level and could be biassing the results. The influence of oestradiol and resProgesterone (random effects) on Hb levels was investigated using linear mixed models (5,6), with participant as a fixed effect. Neither oestradiol (*χ*^2^(1)= 1.241; p=0.265) nor resProgesterone (*χ*^2^(1)= 1.995; p=0.158) significantly contributed to haematocrit variance, suggesting that this was not biasing the OEF and CMRO_2_ results.

*End-tidal CO_2_*

Baseline partial pressures of end-tidal CO_2_ traces were measured from expirations collected using a facemask and an AD Instruments gas analyser and data sampling system (PowerLab®, ADInstruments, Sydney, Australia). This was collected for 20 participants, with 49 total datapoints. In order to examine whether endocrine changes in blood CO_2_ could be driving our perfusion result, 500 seconds of baseline recording were averaged over (median) and linear mixed models used to investigate whether oestradiol or resProgesterone explained a significant amount of variance (using participant as a fixed effect). It was found that neither oestradiol (*χ*^2^(1)= 1.409; p=0.235) nor resProgesterone (*χ*^2^(1)= 1.186; p=0.276) significantly contributed to end-tidal CO_2_ variance.

*Exploratory relationships analysis*

*
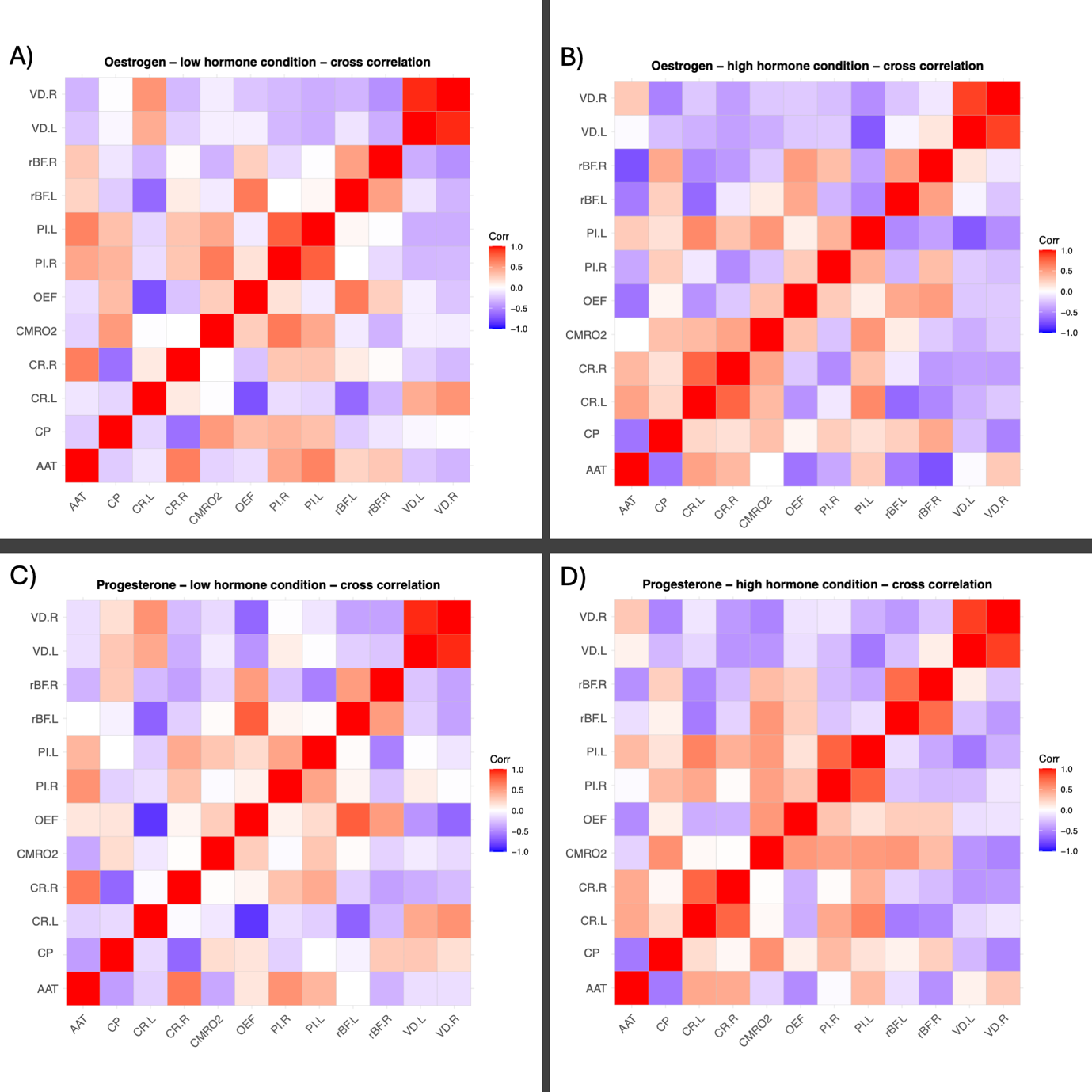
*

*Figure S1 –* A cross-correlation matrix of each condition (low oestrogen, high oestrogen, low progesterone, high progesterone) to be analysed in the principal component analysis. The correlation statistic in a Person’s rho, with more red values indicated more positive correlations, and more blue values indicating more negative correlations. AAT=Arterial arrival time*; CP=cerebral perfusion*; CR.L=Carotid radius left; CR.R=Carotid radius right; CMRO_2_=Cerebral metabolic rate of oxygen; OEF=Oxygen extraction fraction; PI.L=Pulsatility index left; PI.R=Pulsatility index right; rBF.L=Retinal blood flow resistance left; rBF.R=Retinal blood flow resistance right; VD.L=Retinal vessel density left; VD.L=Retinal vessel density left.

*
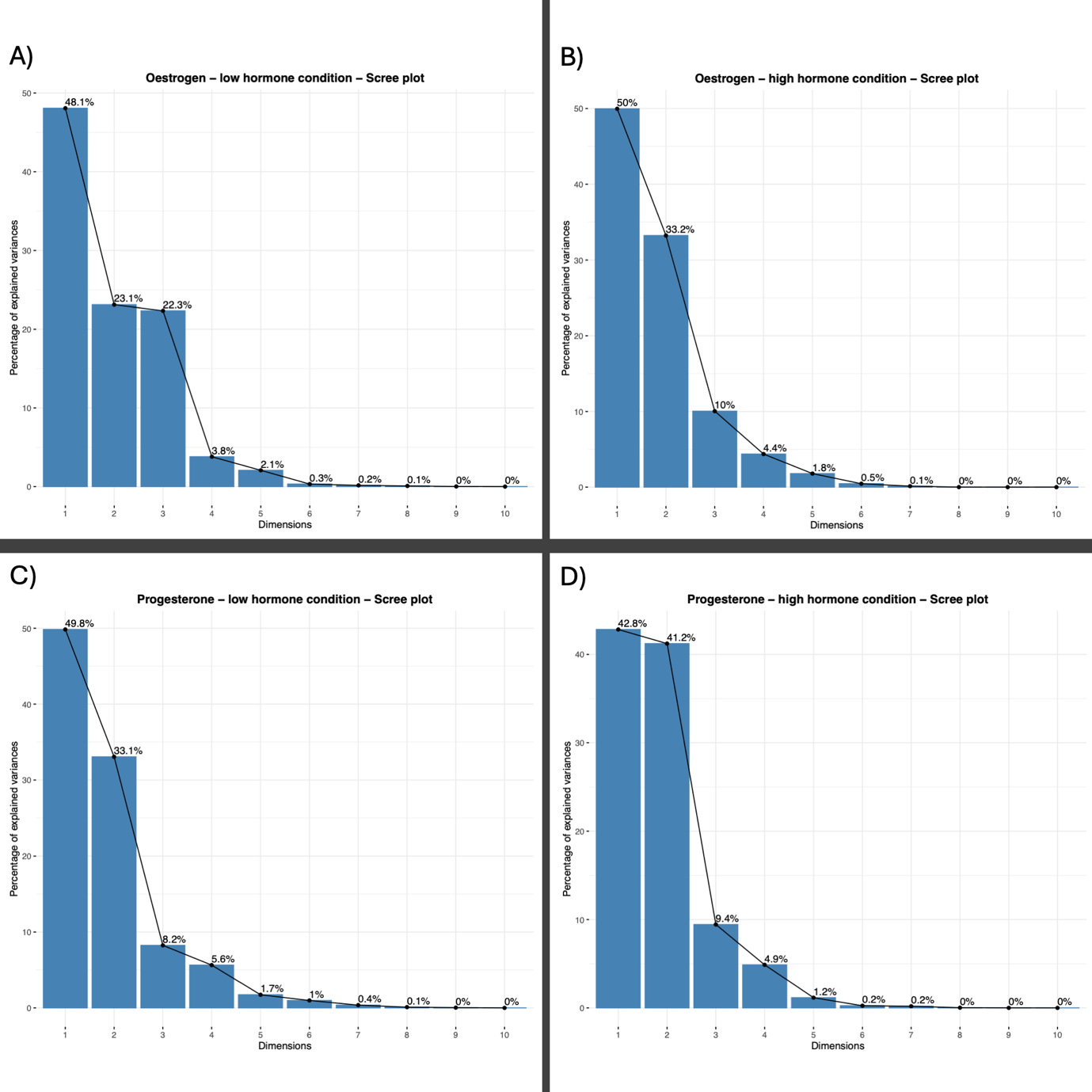
*

*Figure S2 –* Scree plots for each of the four conditions (low oestrogen, high oestrogen, low progesterone, high progesterone) following principal component analysis (PCA), illustrating the amount of variance explained by the generated dimensions/components.

**References**

1. Jenkinson M, Beckmann CF, Behrens TEJ, Woolrich MW, Smith SM. FSL. NeuroImage. 2012 Aug;62(2):782–90.

2. Smith SM, Jenkinson M, Woolrich MW, Beckmann CF, Behrens TEJ, Johansen-Berg H, et al. Advances in functional and structural MR image analysis and implementation as FSL. NeuroImage. 2004 Jan;23:S208–19.

3. Woolrich MW, Jbabdi S, Patenaude B, Chappell M, Makni S, Behrens T, et al. Bayesian analysis of neuroimaging data in FSL. NeuroImage. 2009 Mar;45(1):S173–86.

4. Zhang Y, Brady M, Smith S. Segmentation of brain MR images through a hidden Markov random field model and the expectation-maximization algorithm. IEEE Trans Med Imaging. 2001 Jan;20(1):45–57.

5. Kuznetsova A, Brockhoff PB, Christensen RHB. **lmerTest** Package: Tests in Linear Mixed Effects Models. J Stat Softw [Internet]. 2017 [cited 2023 Oct 9];82(13). Available from: http://www.jstatsoft.org/v82/i13/

6. R core team. R: A language and environment for statistical computing. [Internet]. Vienna, Austria.: R Foundation for Statistical Computing; 2022. Available from: https://www.R-project.org/
